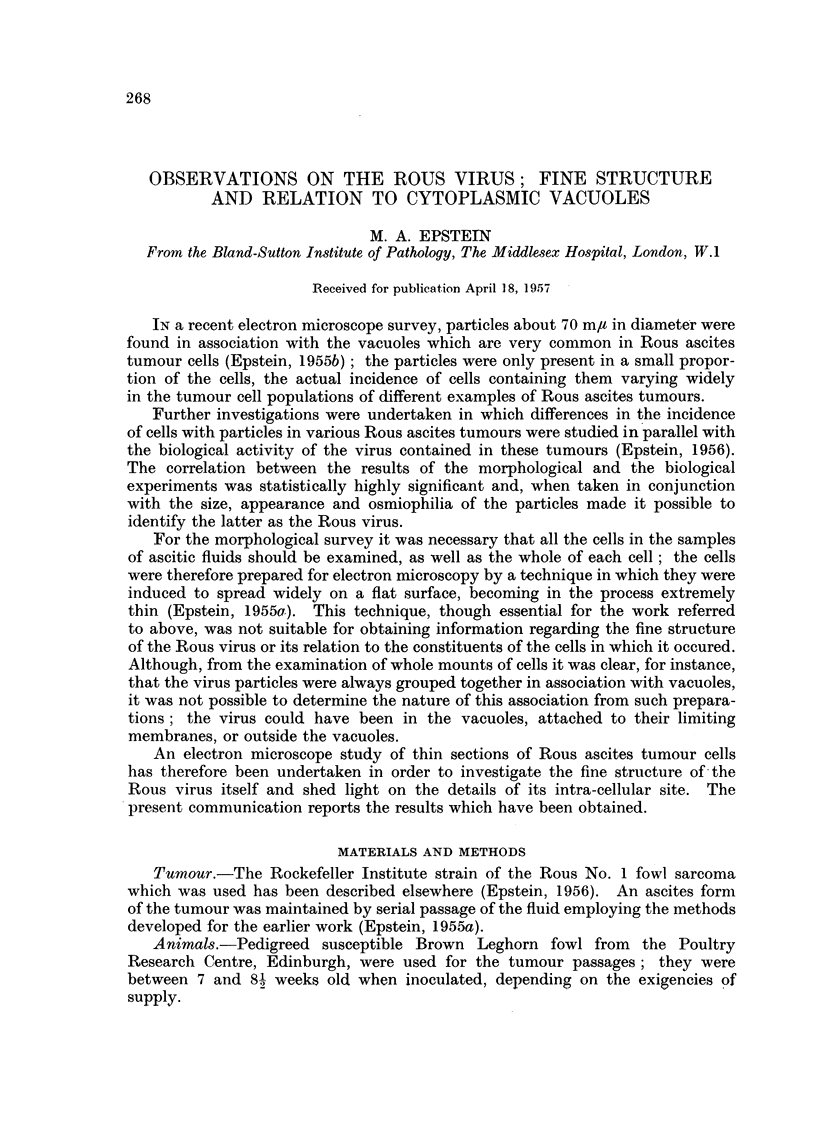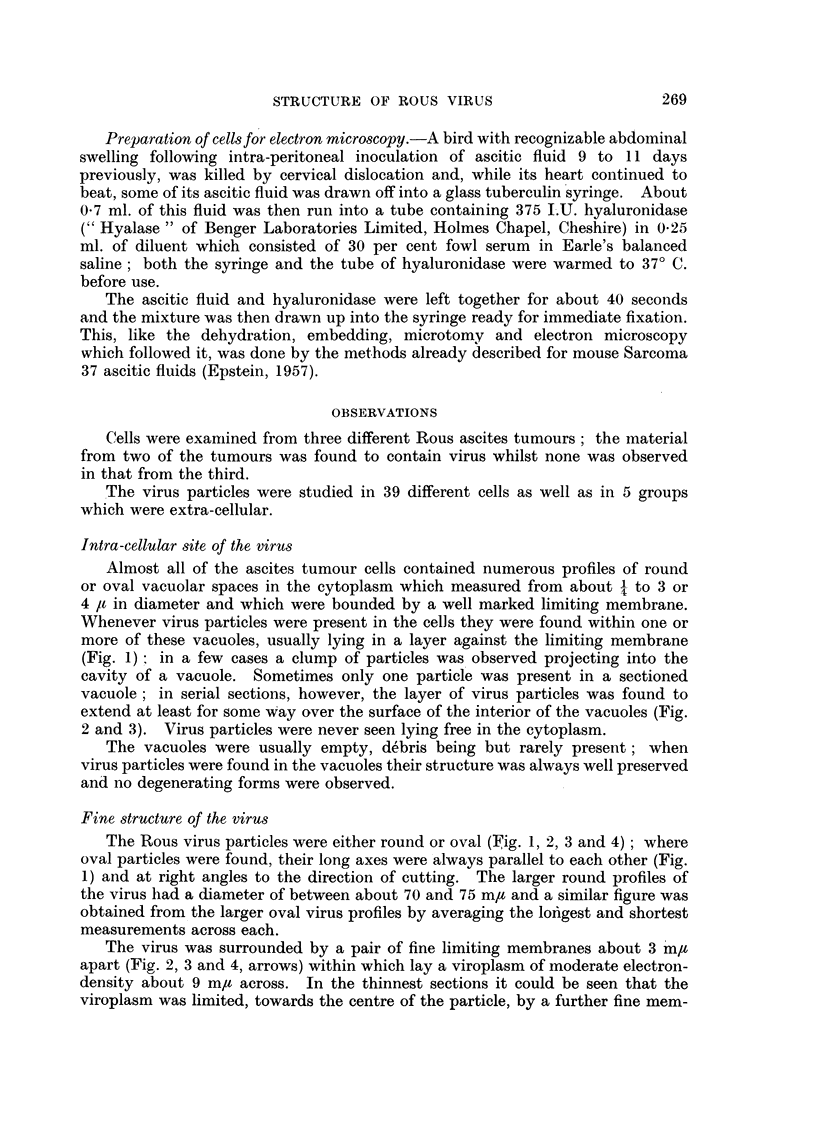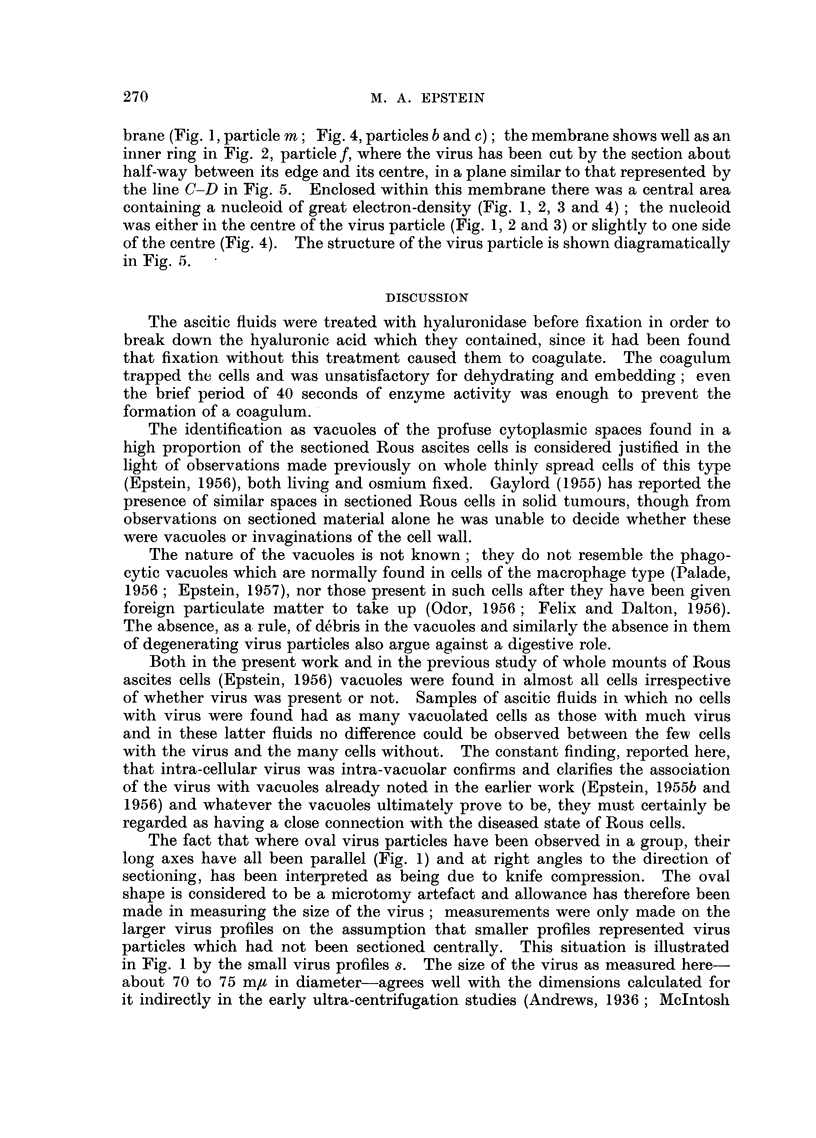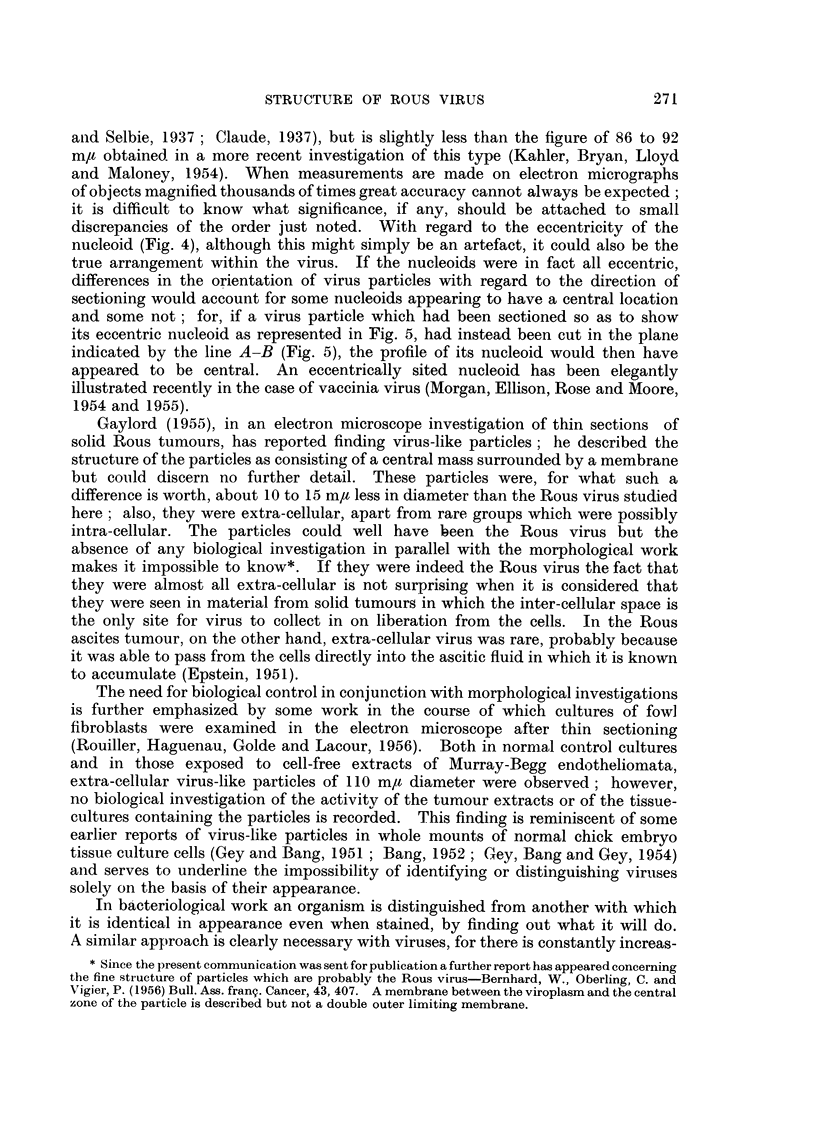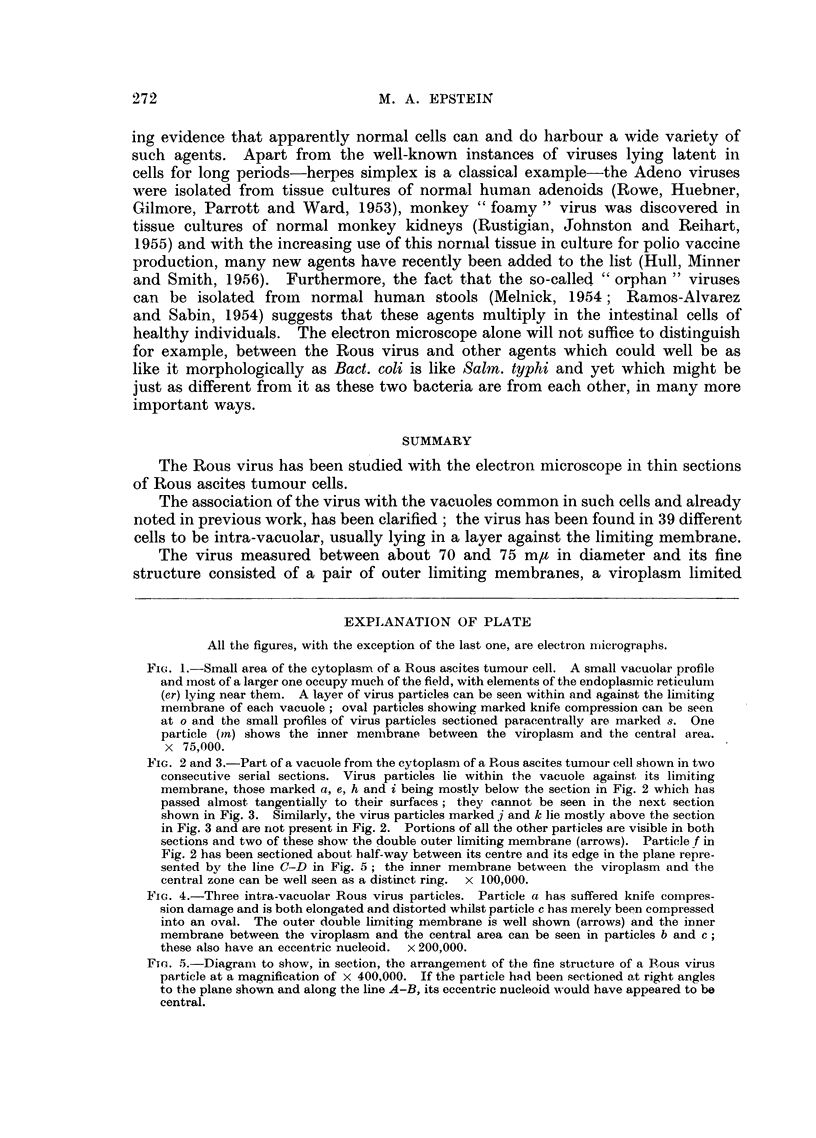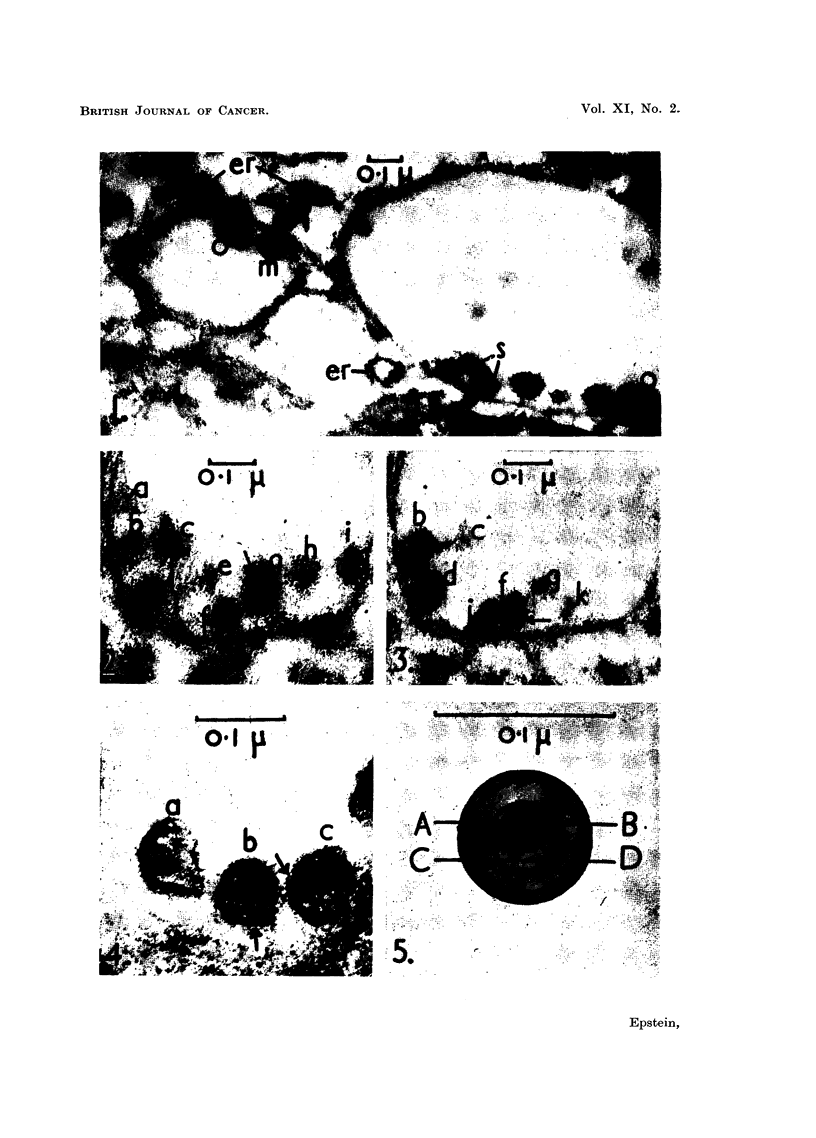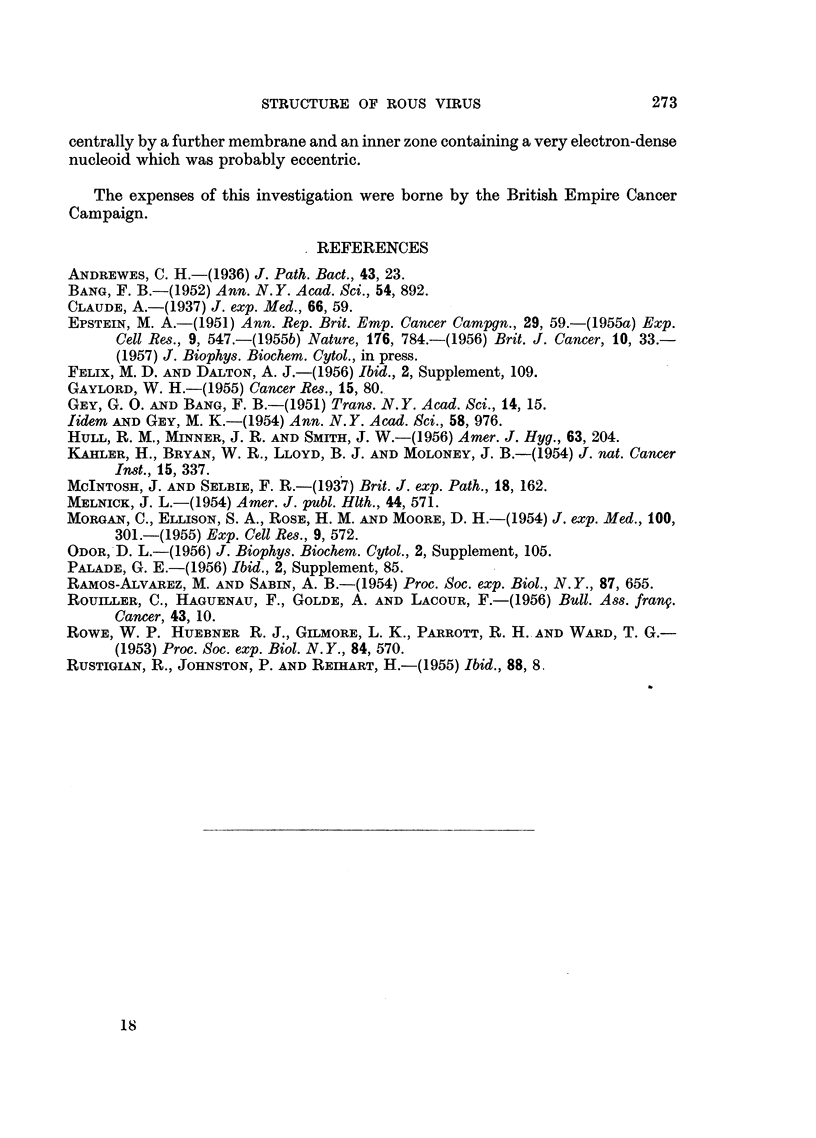# Observations on the Rous Virus; Fine Structure and Relation to Cytoplasmic Vacuoles

**DOI:** 10.1038/bjc.1957.34

**Published:** 1957-06

**Authors:** M. A. Epstein

## Abstract

**Images:**


### OBSERVATIONS ON THE ROUS VIRUS ; FINE STRUCTURE AND RELATION TO CYTOPLASMIC VACUOLES

M. A. EPSTEIN

*From the Bland-Sutton Institute of Pathology, The Middlesex Hospital, London, W.*1

Received for publication April 18, 1957

IN a recent electron microscope survey, particles about 70 m$\mu$ in diameter were found in association with the vacuoles which are very common in Rous ascites tumour cells (Epstein, 1955*b*) ; the particles were only present in a small proportion of the cells, the actual incidence of cells containing them varying widely in the tumour cell populations of different examples of Rous ascites tumours.

Further investigations were undertaken in which differences in the incidence of cells with particles in various Rous ascites tumours were studied in parallel with the biological activity of the virus contained in these tumours (Epstein, 1956). The correlation between the results of the morphological and the biological experiments was statistically highly significant and, when taken in conjunction with the size, appearance and osmiophilia of the particles made it possible to identify the latter as the Rous virus.

For the morphological survey it was necessary that all the cells in the samples of ascitic fluids should be examined, as well as the whole of each cell ; the cells were therefore prepared for electron microscopy by a technique in which they were induced to spread widely on a flat surface, becoming in the process extremely thin (Epstein, 1955*a*). This technique, though essential for the work referred to above, was not suitable for obtaining information regarding the fine structure of the Rous virus or its relation to the constituents of the cells in which it occured. Although, from the examination of whole mounts of cells it was clear, for instance, that the virus particles were always grouped together in association with vacuoles, it was not possible to determine the nature of this association from such preparations ; the virus could have been in the vacuoles, attached to their limiting membranes, or outside the vacuoles.

An electron microscope study of thin sections of Rous ascites tumour cells has therefore been undertaken in order to investigate the fine structure of the Rous virus itself and shed light on the details of its intra-cellular site. The present communication reports the results which have been obtained.

### MATERIALS AND METHODS

*Tumour.*—The Rockefeller Institute strain of the Rous No. 1 fowl sarcoma which was used has been described elsewhere (Epstein, 1956). An ascites form of the tumour was maintained by serial passage of the fluid employing the methods developed for the earlier work (Epstein, 1955*a*).

*Animals.*—Pedigreed susceptible Brown Leghorn fowl from the Poultry Research Centre, Edinburgh, were used for the tumour passages ; they were between 7 and $8\frac{1}{2}$ weeks old when inoculated, depending on the exigencies of supply.

*Preparation of cells for electron microscopy.*—A bird with recognizable abdominal swelling following intra-peritoneal inoculation of ascitic fluid 9 to 11 days previously, was killed by cervical dislocation and, while its heart continued to beat, some of its ascitic fluid was drawn off into a glass tuberculin syringe. About 0·7 ml. of this fluid was then run into a tube containing 375 I.U. hyaluronidase (" Hyalase " of Benger Laboratories Limited, Holmes Chapel, Cheshire) in 0·25 ml. of diluent which consisted of 30 per cent fowl serum in Earle's balanced saline ; both the syringe and the tube of hyaluronidase were warmed to 37° C. before use.

The ascitic fluid and hyaluronidase were left together for about 40 seconds and the mixture was then drawn up into the syringe ready for immediate fixation. This, like the dehydration, embedding, microtomy and electron microscopy which followed it, was done by the methods already described for mouse Sarcoma 37 ascitic fluids (Epstein, 1957).

### OBSERVATIONS

Cells were examined from three different Rous ascites tumours ; the material from two of the tumours was found to contain virus whilst none was observed in that from the third.

The virus particles were studied in 39 different cells as well as in 5 groups which were extra-cellular.

### *Intra-cellular site of the virus*

Almost all of the ascites tumour cells contained numerous profiles of round or oval vacuolar spaces in the cytoplasm which measured from about $\frac{1}{4}$ to 3 or 4 $\mu$ in diameter and which were bounded by a well marked limiting membrane. Whenever virus particles were present in the cells they were found within one or more of these vacuoles, usually lying in a layer against the limiting membrane (Fig. 1) ; in a few cases a clump of particles was observed projecting into the cavity of a vacuole. Sometimes only one particle was present in a sectioned vacuole ; in serial sections, however, the layer of virus particles was found to extend at least for some way over the surface of the interior of the vacuoles (Fig. 2 and 3). Virus particles were never seen lying free in the cytoplasm.

The vacuoles were usually empty, débris being but rarely present ; when virus particles were found in the vacuoles their structure was always well preserved and no degenerating forms were observed.

### *Fine structure of the virus*

The Rous virus particles were either round or oval (Fig. 1, 2, 3 and 4) ; where oval particles were found, their long axes were always parallel to each other (Fig. 1) and at right angles to the direction of cutting. The larger round profiles of the virus had a diameter of between about 70 and 75 m$\mu$ and a similar figure was obtained from the larger oval virus profiles by averaging the longest and shortest measurements across each.

The virus was surrounded by a pair of fine limiting membranes about 3 m$\mu$ apart (Fig. 2, 3 and 4, arrows) within which lay a viroplasm of moderate electron-density about 9 m$\mu$ across. In the thinnest sections it could be seen that the viroplasm was limited, towards the centre of the particle, by a further fine membrane (Fig. 1, particle *m*; Fig. 4, particles *b* and *c*); the membrane shows well as an inner ring in Fig. 2, particle *f*, where the virus has been cut by the section about half-way between its edge and its centre, in a plane similar to that represented by the line *C–D* in Fig. 5. Enclosed within this membrane there was a central area containing a nucleoid of great electron-density (Fig. 1, 2, 3 and 4); the nucleoid was either in the centre of the virus particle (Fig. 1, 2 and 3) or slightly to one side of the centre (Fig. 4). The structure of the virus particle is shown diagramatically in Fig. 5.

### DISCUSSION

The ascitic fluids were treated with hyaluronidase before fixation in order to break down the hyaluronic acid which they contained, since it had been found that fixation without this treatment caused them to coagulate. The coagulum trapped the cells and was unsatisfactory for dehydrating and embedding; even the brief period of 40 seconds of enzyme activity was enough to prevent the formation of a coagulum.

The identification as vacuoles of the profuse cytoplasmic spaces found in a high proportion of the sectioned Rous ascites cells is considered justified in the light of observations made previously on whole thinly spread cells of this type (Epstein, 1956), both living and osmium fixed. Gaylord (1955) has reported the presence of similar spaces in sectioned Rous cells in solid tumours, though from observations on sectioned material alone he was unable to decide whether these were vacuoles or invaginations of the cell wall.

The nature of the vacuoles is not known; they do not resemble the phagocytic vacuoles which are normally found in cells of the macrophage type (Palade, 1956; Epstein, 1957), nor those present in such cells after they have been given foreign particulate matter to take up (Odor, 1956; Felix and Dalton, 1956). The absence, as a rule, of débris in the vacuoles and similarly the absence in them of degenerating virus particles also argue against a digestive role.

Both in the present work and in the previous study of whole mounts of Rous ascites cells (Epstein, 1956) vacuoles were found in almost all cells irrespective of whether virus was present or not. Samples of ascitic fluids in which no cells with virus were found had as many vacuolated cells as those with much virus and in these latter fluids no difference could be observed between the few cells with the virus and the many cells without. The constant finding, reported here, that intra-cellular virus was intra-vacuolar confirms and clarifies the association of the virus with vacuoles already noted in the earlier work (Epstein, 1955*b* and 1956) and whatever the vacuoles ultimately prove to be, they must certainly be regarded as having a close connection with the diseased state of Rous cells.

The fact that where oval virus particles have been observed in a group, their long axes have all been parallel (Fig. 1) and at right angles to the direction of sectioning, has been interpreted as being due to knife compression. The oval shape is considered to be a microtomy artefact and allowance has therefore been made in measuring the size of the virus; measurements were only made on the larger virus profiles on the assumption that smaller profiles represented virus particles which had not been sectioned centrally. This situation is illustrated in Fig. 1 by the small virus profiles *s*. The size of the virus as measured here—about 70 to 75 m$\mu$ in diameter—agrees well with the dimensions calculated for it indirectly in the early ultra-centrifugation studies (Andrews, 1936; McIntosh and Selbie, 1937 ; Claude, 1937), but is slightly less than the figure of 86 to 92 m$\mu$ obtained in a more recent investigation of this type (Kahler, Bryan, Lloyd and Maloney, 1954). When measurements are made on electron micrographs of objects magnified thousands of times great accuracy cannot always be expected ; it is difficult to know what significance, if any, should be attached to small discrepancies of the order just noted. With regard to the eccentricity of the nucleoid (Fig. 4), although this might simply be an artefact, it could also be the true arrangement within the virus. If the nucleoids were in fact all eccentric, differences in the orientation of virus particles with regard to the direction of sectioning would account for some nucleoids appearing to have a central location and some not ; for, if a virus particle which had been sectioned so as to show its eccentric nucleoid as represented in Fig. 5, had instead been cut in the plane indicated by the line *A–B* (Fig. 5), the profile of its nucleoid would then have appeared to be central. An eccentrically sited nucleoid has been elegantly illustrated recently in the case of vaccinia virus (Morgan, Ellison, Rose and Moore, 1954 and 1955).

Gaylord (1955), in an electron microscope investigation of thin sections of solid Rous tumours, has reported finding virus-like particles ; he described the structure of the particles as consisting of a central mass surrounded by a membrane but could discern no further detail. These particles were, for what such a difference is worth, about 10 to 15 m$\mu$ less in diameter than the Rous virus studied here ; also, they were extra-cellular, apart from rare groups which were possibly intra-cellular. The particles could well have been the Rous virus but the absence of any biological investigation in parallel with the morphological work makes it impossible to know*. If they were indeed the Rous virus the fact that they were almost all extra-cellular is not surprising when it is considered that they were seen in material from solid tumours in which the inter-cellular space is the only site for virus to collect in on liberation from the cells. In the Rous ascites tumour, on the other hand, extra-cellular virus was rare, probably because it was able to pass from the cells directly into the ascitic fluid in which it is known to accumulate (Epstein, 1951).

The need for biological control in conjunction with morphological investigations is further emphasized by some work in the course of which cultures of fowl fibroblasts were examined in the electron microscope after thin sectioning (Rouiller, Haguenau, Golde and Lacour, 1956). Both in normal control cultures and in those exposed to cell-free extracts of Murray-Begg endotheliomata, extra-cellular virus-like particles of 110 m$\mu$ diameter were observed ; however, no biological investigation of the activity of the tumour extracts or of the tissue-cultures containing the particles is recorded. This finding is reminiscent of some earlier reports of virus-like particles in whole mounts of normal chick embryo tissue culture cells (Gey and Bang, 1951 ; Bang, 1952 ; Gey, Bang and Gey, 1954) and serves to underline the impossibility of identifying or distinguishing viruses solely on the basis of their appearance.

In bacteriological work an organism is distinguished from another with which it is identical in appearance even when stained, by finding out what it will do. A similar approach is clearly necessary with viruses, for there is constantly increas-

* Since the present communication was sent for publication a further report has appeared concerning the fine structure of particles which are probably the Rous virus—Bernhard, W., Oberling, C. and Vigier, P. (1956) Bull. Ass. franç. Cancer, 43, 407. A membrane between the viroplasm and the central zone of the particle is described but not a double outer limiting membrane.

ing evidence that apparently normal cells can and do harbour a wide variety of such agents. Apart from the well-known instances of viruses lying latent in cells for long periods—herpes simplex is a classical example—the Adeno viruses were isolated from tissue cultures of normal human adenoids (Rowe, Huebner, Gilmore, Parrott and Ward, 1953), monkey " foamy " virus was discovered in tissue cultures of normal monkey kidneys (Rustigian, Johnston and Reihart, 1955) and with the increasing use of this normal tissue in culture for polio vaccine production, many new agents have recently been added to the list (Hull, Minner and Smith, 1956). Furthermore, the fact that the so-called " orphan " viruses can be isolated from normal human stools (Melnick, 1954; Ramos-Alvarez and Sabin, 1954) suggests that these agents multiply in the intestinal cells of healthy individuals. The electron microscope alone will not suffice to distinguish for example, between the Rous virus and other agents which could well be as like it morphologically as *Bact. coli* is like *Salm. typhi* and yet which might be just as different from it as these two bacteria are from each other, in many more important ways.

### SUMMARY

The Rous virus has been studied with the electron microscope in thin sections of Rous ascites tumour cells.

The association of the virus with the vacuoles common in such cells and already noted in previous work, has been clarified; the virus has been found in 39 different cells to be intra-vacuolar, usually lying in a layer against the limiting membrane.

The virus measured between about 70 and 75 m$\mu$ in diameter and its fine structure consisted of a pair of outer limiting membranes, a viroplasm limited

---

### EXPLANATION OF PLATE

All the figures, with the exception of the last one, are electron micrographs.

FIG. 1.—Small area of the cytoplasm of a Rous ascites tumour cell. A small vacuolar profile and most of a larger one occupy much of the field, with elements of the endoplasmic reticulum (*er*) lying near them. A layer of virus particles can be seen within and against the limiting membrane of each vacuole; oval particles showing marked knife compression can be seen at *o* and the small profiles of virus particles sectioned paracentrally are marked *s*. One particle (*m*) shows the inner membrane between the viroplasm and the central area. × 75,000.

FIG. 2 and 3.—Part of a vacuole from the cytoplasm of a Rous ascites tumour cell shown in two consecutive serial sections. Virus particles lie within the vacuole against its limiting membrane, those marked *a*, *e*, *h* and *i* being mostly below the section in Fig. 2 which has passed almost tangentially to their surfaces; they cannot be seen in the next section shown in Fig. 3. Similarly, the virus particles marked *j* and *k* lie mostly above the section in Fig. 3 and are not present in Fig. 2. Portions of all the other particles are visible in both sections and two of these show the double outer limiting membrane (arrows). Particle *f* in Fig. 2 has been sectioned about half-way between its centre and its edge in the plane represented by the line *C–D* in Fig. 5; the inner membrane between the viroplasm and the central zone can be well seen as a distinct ring. × 100,000.

FIG. 4.—Three intra-vacuolar Rous virus particles. Particle *a* has suffered knife compression damage and is both elongated and distorted whilst particle *c* has merely been compressed into an oval. The outer double limiting membrane is well shown (arrows) and the inner membrane between the viroplasm and the central area can be seen in particles *b* and *c*; these also have an eccentric nucleoid. ×200,000.

FIG. 5.—Diagram to show, in section, the arrangement of the fine structure of a Rous virus particle at a magnification of × 400,000. If the particle had been sectioned at right angles to the plane shown and along the line *A–B*, its eccentric nucleoid would have appeared to be central.

er
0·1 μ
o
m
er
s
j
o
1.

0·1 μ
a
b
c
d
e
g
h
i
f
2.

0·1 μ
b
c
d
f
g
k
j
3.

0·1 μ
a
b
c
4.

0·1 μ
A
B
C
D
5.

centrally by a further membrane and an inner zone containing a very electron-dense nucleoid which was probably eccentric.

The expenses of this investigation were borne by the British Empire Cancer Campaign.

### REFERENCES

ANDREWES, C. H.—(1936) *J. Path. Bact.*, **43**, 23.
BANG, F. B.—(1952) *Ann. N.Y. Acad. Sci.*, **54**, 892.
CLAUDE, A.—(1937) *J. exp. Med.*, **66**, 59.
EPSTEIN, M. A.—(1951) *Ann. Rep. Brit. Emp. Cancer Campgn.*, **29**, 59.—(1955*a*) *Exp. Cell Res.*, **9**, 547.—(1955*b*) *Nature*, **176**, 784.—(1956) *Brit. J. Cancer*, **10**, 33.—(1957) *J. Biophys. Biochem. Cytol.*, in press.
FELIX, M. D. AND DALTON, A. J.—(1956) *Ibid.*, **2**, Supplement, 109.
GAYLORD, W. H.—(1955) *Cancer Res.*, **15**, 80.
GEY, G. O. AND BANG, F. B.—(1951) *Trans. N.Y. Acad. Sci.*, **14**, 15.
*Iidem* AND GEY, M. K.—(1954) *Ann. N.Y. Acad. Sci.*, **58**, 976.
HULL, R. M., MINNER, J. R. AND SMITH, J. W.—(1956) *Amer. J. Hyg.*, **63**, 204.
KAHLER, H., BRYAN, W. R., LLOYD, B. J. AND MOLONEY, J. B.—(1954) *J. nat. Cancer Inst.*, **15**, 337.
McINTOSH, J. AND SELBIE, F. R.—(1937) *Brit. J. exp. Path.*, **18**, 162.
MELNICK, J. L.—(1954) *Amer. J. publ. Hlth.*, **44**, 571.
MORGAN, C., ELLISON, S. A., ROSE, H. M. AND MOORE, D. H.—(1954) *J. exp. Med.*, **100**, 301.—(1955) *Exp. Cell Res.*, **9**, 572.
ODOR, D. L.—(1956) *J. Biophys. Biochem. Cytol.*, **2**, Supplement, 105.
PALADE, G. E.—(1956) *Ibid.*, **2**, Supplement, 85.
RAMOS-ALVAREZ, M. AND SABIN, A. B.—(1954) *Proc. Soc. exp. Biol., N.Y.*, **87**, 655.
ROUILLER, C., HAGUENAU, F., GOLDE, A. AND LACOUR, F.—(1956) *Bull. Ass. franç. Cancer*, **43**, 10.
ROWE, W. P. HUEBNER R. J., GILMORE, L. K., PARROTT, R. H. AND WARD, T. G.—(1953) *Proc. Soc. exp. Biol. N.Y.*, **84**, 570.
RUSTIGIAN, R., JOHNSTON, P. AND REIHART, H.—(1955) *Ibid.*, **88**, 8.